# Crystal structure of aqua­chlorido­bis­(2-eth­oxy-6-formyl­phenolato-κ^2^ *O* ^1^,*O* ^6^)iron(III) aceto­nitrile hemisolvate

**DOI:** 10.1107/S1600536814021205

**Published:** 2014-10-04

**Authors:** Xi-Fu Jiang, Ru-Xia Zhao, Shu-Hua Zhang

**Affiliations:** a College of Chemistry and Bioengineering, Guilin University of Technology, 541004, People’s Republic of China

**Keywords:** crystal structure, solvothermal synthesis, Fe(III) complex, dimer, hydrogen bonding.

## Abstract

In the title compound, [Fe(*L*)_2_Cl(H_2_O)]·0.5CH_3_CN, (H*L* is 3-eth­oxy-2-hy­droxy-benzaldehyde, C_9_H_10_O_3_), there are two independent complex mol­ecules and one aceto­nitrile solvent mol­ecule in the asymmetric unit. In each complex mol­ecule, the Fe^III^ ion has a distorted O_5_Cl octa­hedral coordination environment defined by two bidentate 2-eth­oxy-6-formyl­phenolato ligands, one Cl atom and one water mol­ecule. In the crystal, O—H⋯O hydrogen bonds link the two independent mol­ecules to form a dimer. The solvent mol­ecule is linked to the complex mol­ecule by a weak C—H⋯O hydrogen bond. Further weak C—H⋯O inter­actions along with weak C—H⋯Cl hydrogen bonds link the components into chains parallel to [001].

## Abstract 2

In both complex mol­ecules in the asymmetric unit, the Fe^III^ ion has a distorted O_5_Cl octa­hedral coordination environment defined by two bidentate 2-eth­oxy-6-formyl­phenolato ligands, one Cl atom and one water mol­ecule. In the crystal, O—H⋯O hydrogen bonds link the two independent mol­ecules to form a dimer while the solvent mol­ecule is linked to the complex mol­ecule by a weak C—H⋯O hydrogen bond. Further weak C—H⋯O inter­actions along with weak C—H⋯Cl hydrogen bonds link the components into chains parallel to [001].

## Chemical context   

Metal complexes containing the 2-hy­droxy-benzaldehyde ramification are one of the most fundamental chelating systems in coordination chemistry. Their inter­esting chemical and physical properties and their wide-ranging applications in numerous scientific areas have been explored widely (Han 2008 ▶; Ghelenji *et al.*, 2011 ▶; Kia *et al.*, 2010 ▶; Zhang *et al.*, 2013 ▶, 2014*a* ▶,*b* ▶; Zhao *et al.*, 2014 ▶). During the last few years, we have investigated the chemistry of 3*d* metal complexes of 2-hy­droxy-benzaldehyde ramification ligands with the aim of preparing mono- and heterometallic polynuclear clusters or polymers (Zhang *et al.*, 2011 ▶, 2013 ▶, 2014*a* ▶,*b* ▶; Zhao *et al.*, 2014 ▶).

Recently, we have investigated the coordination behavior of the tridentate 2-hy­droxy-benzaldehyde ramification ligand 3-eth­oxy-2-hy­droxy-benzaldehyde and reported two heterometallic polymers [ZnNa(ehbd)_2_(N_3_)]_*n*_ and [Cu_3_Na_2_(ehbd)_2_(N_3_)_6_]_*n*_ (ehbd is the 2-hydroxy-3-ethoxy-benzaldehyde anion) (Zhang *et al.*, 2014*b* ▶) and a cubane cluster [Ni_4_(*μ*_3_-OMe)_4_(heb)_4_(MeOH)_1.05_(H_2_O)_2.95_] (heb is the 2-hydroxy-3-ethoxy-benzaldehyde anion) (Zhang *et al.*, 2011 ▶). The polymers [ZnNa(ehbd)_2_(N_3_)]_*n*_ and [Cu_3_Na_2_(ehbd)_2_(N_3_)_6_]_*n*_ were prepared by room-temperature synthesis and the cubane cluster [Ni_4_(*μ*_3_-OMe)_4_(heb)_4_(MeOH)_1.05_(H_2_O)_2.95_] was prepared by solvothermal synthesis. Those complexes display dominant ferromagnetic inter­actions between metal ions.

The title compound, [Fe(*L*)_2_Cl(H_2_O)]·0.5CH_3_CN (H*L* = C_9_H_10_O_3_), was prepared similarly to the cubane cluster [Ni_4_(*μ*_3_-OMe)_4_(heb)_4_(MeOH)_1.05_(H_2_O)_2.95_] (Zhang *et al.*, 2011 ▶) except that Ni(ClO_4_)·6H_2_O was replaced by FeCl_3_·6H_2_O in an attempt to prepare a cubane-type iron cluster. The crystals obtained, however, were those of the title mononuclear Fe^III^ complex.
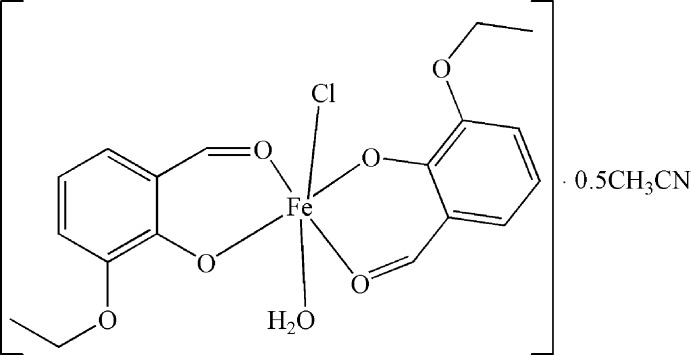


## Structural commentary   

**Figure 1:**
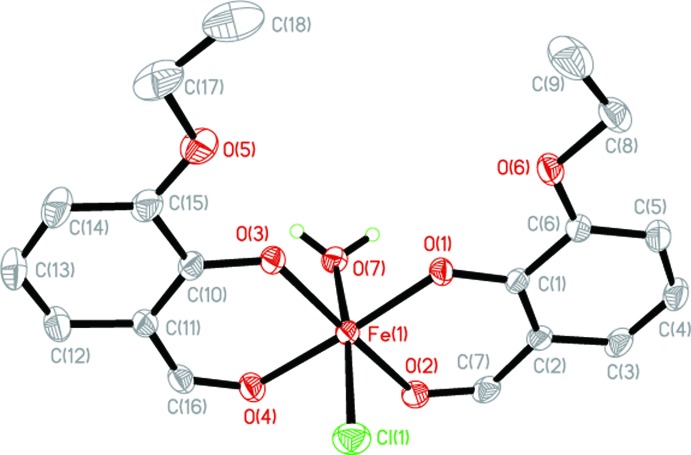
The mol­ecular structure of one complex mol­ecule of the title compound showing displacement ellipsoids drawn at the 30% probability level for non-H atoms. H atoms bonded to C atoms and the solvent mol­ecule are not shown.

The asymmetric unit of the title compound consists of two neutral [Fe(*L*)_2_Cl(H_2_O)] mol­ecules and a aceto­nitrile solvent mol­ecule. One of the independent mol­ecules is shown in Fig. 1 ▶. Each Fe^III^ ion is coordinated by four O atoms from two different *L^−^* ligands, one Cl^−^ ion and one terminal water mol­ecule, forming a distorted octa­hedral geometry. The Fe—O bond lengths are in the range 1.909 (2)–2.157 (2) Å (Table 1 ▶), while the Fe—Cl distances are 2.299 (1) and 2.301 (1) Å. The *trans*-angles at the Fe^III^ atom lie in the range 169.4 (1)–171.4 (1)°, the *cis*-angles vary from 81.6 (1) to 99.9 (1)°. The *L^−^* ligand displays a *μ*_1_:*κ* ^1^:*κ* ^1^ coordination mode, which is the same as that of [Ni_4_(*μ*_3_-OMe)_4_(heb)_4_(MeOH)_1.05_(H_2_O)_2.95_] (Zhang *et al.*, 2011 ▶) but the coordination mode is different from the that in [Cu_3_Na_2_(ehbd)_2_(N_3_)_6_]_*n*_ (Zhang *et al.*, 2014*b* ▶) in which the ehbd^−^ ligand displays a penta­dentate *μ*_3_:*κ* ^2^:*κ* ^2^:*κ* ^1^ coordination mode.

## Supra­molecular features   

**Figure 2:**
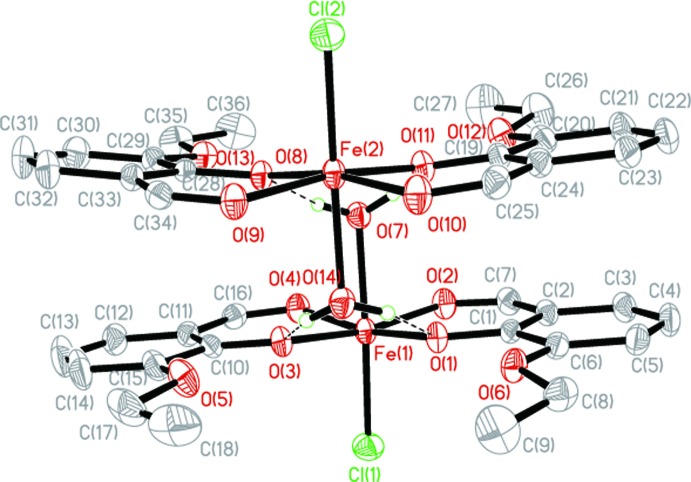
The dimer structure showing displacement ellipsoids drawn at the 30% probability level for non-H atoms. Hydrogen bonds are shown as dashed lines. H atoms bonded to C atoms and the solvent mol­ecule are not shown.

**Figure 3:**
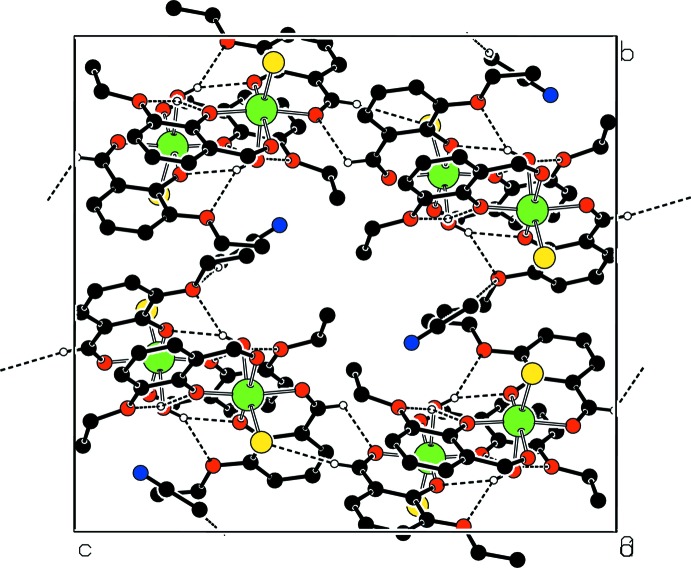
Part of the crystal structure with hydrogen bonds drawn as dashed lines.

In the crystal, O—H⋯O hydrogen bonds link the two independent mol­ecules to form a dimer (Table 2 ▶, Fig. 2 ▶). All –OH group H atoms act as donors for two acceptor-O atoms, forming 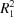
(5) and 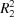
(6) graph-set motifs. A π–π inter­action within the dimer with a *Cg*1⋯*Cg*2 distance of 3.575 (1)Å is observed, where *Cg*1 and *Cg*2 are the centroids defined by ring atoms C1–C6 and C19–C24, respectively. The solvent mol­ecule is linked to the complex mol­ecule by a weak C—H⋯O hydrogen bond. Further weak C—H⋯O inter­action along with weak C—H⋯Cl hydrogen bonds link the components into chains parallel to [001] (Fig. 3 ▶).

## Synthesis and crystallization   

A mixture of FeCl_3_·6H_2_O (0.135 g, 0.5 mmol), 3-eth­oxy-2-hy­droxy-benzaldehyde (0.168 g, 1 mmol), methanol (5 mL) and aceto­nitrile (5 mL), with a pH adjusted to 7.5 by addition of tri­ethyl­amine, was poured into a Teflon-lined autoclave (15 mL) and then heated at 413K for 3 days. Black crystals of the title compound were collected by filtration, washed with methanol and dried in air. Phase pure crystals were obtained by manual separation (yield: 124 mg, *ca* 54% based on Fe).

## Refinement   

Crystal data, data collection and structure refinement details are summarized in Table 3 ▶. All H atoms bonded to C atoms were positioned geometrically and refined as riding atoms, with C—H distances of 0.93 (aromatic), 0.96 (CH_2_) or 0.97 Å (CH_3_) with *U*_iso_(H) = 1.2*U*_eq_(C) or 1.5*U*_eq_(C_meth­yl_). H atoms bonded to O atoms were included with O—H = 0.84–0.85 Å and with *U*_iso_(H) = 1.5*U*_eq_(O).

## Supplementary Material

Crystal structure: contains datablock(s) I. DOI: 10.1107/S1600536814021205/lh5728sup1.cif

Structure factors: contains datablock(s) I. DOI: 10.1107/S1600536814021205/lh5728Isup2.hkl

Supporting information file. DOI: 10.1107/S1600536814021205/lh5728Isup3.cdx

CCDC reference: 1025672

Additional supporting information:  crystallographic information; 3D view; checkCIF report

## Figures and Tables

**Table 1:** Selected bond lengths ()

Fe1O1	1.9088(17)	Fe2O10	1.9181(16)
Fe1O3	1.9296(16)	Fe2O8	1.9343(17)
Fe1O2	2.0447(17)	Fe2O9	2.0551(17)
Fe1O4	2.0719(18)	Fe2O11	2.0763(18)
Fe1O7	2.1573(18)	Fe2O14	2.1379(18)

**Table 2:** Hydrogen-bond geometry (, )

*D*H*A*	*D*H	H*A*	*D* *A*	*D*H*A*
O7H7O10	0.85	2.22	2.887(2)	136
O7H7O12	0.85	2.25	3.027(3)	153
O14H14*A*O3	0.85	2.13	2.862(2)	145
O14H14*A*O5	0.85	2.28	3.008(3)	143
O7H7*B*O8	0.84	2.19	2.896(2)	142
O7H7*B*O13	0.84	2.33	3.063(2)	146
O14H14*B*O1	0.84	2.23	2.908(2)	139
O14H14*B*O6	0.84	2.28	3.026(2)	149
C7H7*A*Cl2^i^	0.93	2.80	3.724(3)	171
C34H34O2^ii^	0.93	2.57	3.014(3)	110
C37H37*C*O6^iii^	0.96	2.58	3.506(5)	162

Symmetry codes: (i) 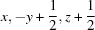; (ii) 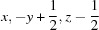; (iii) 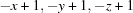.

**Table 3:** Experimental details

Crystal data	
Chemical formula	[Fe(C_9_H_9_O_3_)_2_Cl(H_2_O)]0.5C_2_H_3_N
*M*_r_	460.17
Crystal system, space group	Monoclinic, *P*2_1_/*c*
Temperature (K)	293
*a*, *b*, *c* ()	11.8565(4), 18.0786(5), 20.5785(6)
()	105.981(3)
*V* (^3^)	4240.5(2)
*Z*	8
Radiation type	Mo *K*
(mm^1^)	0.88
Crystal size (mm)	0.24 0.22 0.19

Data collection	
Diffractometer	SuperNova, Single source at offset, Eos
Absorption correction	Multi-scan (*CrysAlis RED*; Agilent, 2012 ▶)
*T*_min_, *T*_max_	0.811, 0.848
No. of measured, independent and observed [*I* > 2(*I*)] reflections	17797, 7544, 6145
*R*_int_	0.022
(sin /)_max_ (^1^)	0.597

Refinement	
*R*[*F* ^2^ > 2(*F* ^2^)], *wR*(*F* ^2^), *S*	0.037, 0.097, 1.00
No. of reflections	7544
No. of parameters	517
H-atom treatment	H-atom parameters constrained
_max_, _min_ (e ^3^)	0.27, 0.24

Computer programs: *CrysAlis PRO* (Agilent, 2012 ▶), *SHELXS97*, *SHELXL97* and *SHELXTL* (Sheldrick, 2008 ▶), *PLATON* (Spek, 2009 ▶) and *OLEX2* (Dolomanov *et al.*, 2009 ▶).

## supplementary crystallographic information

### Crystal data

**Table:**

[Fe(C_9_H_9_O_3_)_2_Cl(H_2_O)]·0.5C_2_H_3_N	*F*(000) = 1904
*M**_r_* = 460.17	*D*_x_ = 1.442 Mg m^−^^3^
Monoclinic, *P*2_1_/*c*	Mo *K*α radiation, λ = 0.71073 Å
*a* = 11.8565 (4) Å	Cell parameters from 8118 reflections
*b* = 18.0786 (5) Å	θ = 3.6–28.5°
*c* = 20.5785 (6) Å	µ = 0.88 mm^−^^1^
β = 105.981 (3)°	*T* = 293 K
*V* = 4240.5 (2) Å^3^	Block, black
*Z* = 8	0.24 × 0.22 × 0.19 mm

### Data collection

**Table:**

SuperNova, Single source at offset, Eos diffractometer	7544 independent reflections
Radiation source: fine-focus sealed tube	6145 reflections with *I* > 2σ(*I*)
Graphite monochromator	*R*_int_ = 0.022
Detector resolution: 0 pixels mm^-1^	θ_max_ = 25.1°, θ_min_ = 2.9°
ω scans	*h* = −11→14
Absorption correction: multi-scan (*CrysAlis RED*; Agilent, 2012)	*k* = −21→15
*T*_min_ = 0.811, *T*_max_ = 0.848	*l* = −24→24
17797 measured reflections	

### Refinement

**Table:**

Refinement on *F*^2^	Primary atom site location: structure-invariant direct methods
Least-squares matrix: full	Secondary atom site location: difference Fourier map
*R*[*F*^2^ > 2σ(*F*^2^)] = 0.037	Hydrogen site location: inferred from neighbouring sites
*wR*(*F*^2^) = 0.097	H-atom parameters constrained
*S* = 1.00	*w* = 1/[σ^2^(*F*_o_^2^) + (0.0404*P*)^2^ + 2.8341*P*] where *P* = (*F*_o_^2^ + 2*F*_c_^2^)/3
7544 reflections	(Δ/σ)_max_ = 0.001
517 parameters	Δρ_max_ = 0.27 e Å^−^^3^
0 restraints	Δρ_min_ = −0.24 e Å^−^^3^

### Special details

**Table:**

Geometry. All e.s.d.'s (except the e.s.d. in the dihedral angle between two l.s. planes) are estimated using the full covariance matrix. The cell e.s.d.'s are taken into account individually in the estimation of e.s.d.'s in distances, angles and torsion angles; correlations between e.s.d.'s in cell parameters are only used when they are defined by crystal symmetry. An approximate (isotropic) treatment of cell e.s.d.'s is used for estimating e.s.d.'s involving l.s. planes.
Refinement. Refinement of *F*^2^ against ALL reflections. The weighted *R*-factor *wR* and goodness of fit *S* are based on *F*^2^, conventional *R*-factors *R* are based on *F*, with *F* set to zero for negative *F*^2^. The threshold expression of *F*^2^ > σ(*F*^2^) is used only for calculating *R*-factors(gt) *etc*. and is not relevant to the choice of reflections for refinement. *R*-factors based on *F*^2^ are statistically about twice as large as those based on *F*, and *R*- factors based on ALL data will be even larger.

### Fractional atomic coordinates and isotropic or equivalent isotropic displacement parameters (Å^2^)

**Table:**

	*x*	*y*	*z*	*U*_iso_*/*U*_eq_	
C1	0.4723 (2)	0.43070 (13)	0.87478 (12)	0.0313 (5)	
C2	0.5036 (2)	0.41391 (14)	0.94458 (12)	0.0361 (6)	
C3	0.6098 (2)	0.44226 (16)	0.98744 (14)	0.0467 (7)	
H3	0.6308	0.4311	1.0333	0.056*	
C4	0.6807 (2)	0.48534 (17)	0.96195 (15)	0.0511 (8)	
H4	0.7501	0.5036	0.9905	0.061*	
C5	0.6504 (2)	0.50250 (15)	0.89322 (14)	0.0457 (7)	
H5	0.6999	0.5323	0.8764	0.055*	
C6	0.5483 (2)	0.47596 (14)	0.84989 (13)	0.0357 (6)	
C7	0.4316 (2)	0.37029 (15)	0.97413 (12)	0.0411 (6)	
H7A	0.4588	0.3627	1.0205	0.049*	
C8	0.5749 (3)	0.54206 (16)	0.75438 (15)	0.0500 (7)	
H8A	0.6522	0.5229	0.7564	0.060*	
H8B	0.5843	0.5876	0.7802	0.060*	
C9	0.5078 (3)	0.5565 (2)	0.68265 (17)	0.0771 (11)	
H9A	0.4918	0.5104	0.6587	0.116*	
H9B	0.5534	0.5876	0.6617	0.116*	
H9C	0.4352	0.5807	0.6815	0.116*	
C10	0.0680 (2)	0.31538 (14)	0.71813 (12)	0.0356 (6)	
C11	−0.0010 (2)	0.27358 (15)	0.74985 (14)	0.0392 (6)	
C12	−0.1098 (3)	0.24408 (18)	0.71145 (17)	0.0557 (8)	
H12	−0.1553	0.2164	0.7328	0.067*	
C13	−0.1476 (3)	0.2559 (2)	0.64441 (18)	0.0737 (11)	
H13	−0.2191	0.2363	0.6198	0.088*	
C14	−0.0808 (3)	0.2970 (2)	0.61172 (16)	0.0684 (10)	
H14	−0.1079	0.3044	0.5653	0.082*	
C15	0.0254 (2)	0.32719 (17)	0.64725 (14)	0.0473 (7)	
C16	0.0336 (2)	0.25793 (14)	0.82021 (14)	0.0413 (6)	
H16	−0.0186	0.2297	0.8363	0.050*	
C17	0.0579 (3)	0.3882 (2)	0.55026 (15)	0.0729 (11)	
H17A	−0.0163	0.4141	0.5415	0.088*	
H17B	0.0466	0.3441	0.5223	0.088*	
C18	0.1481 (4)	0.4368 (2)	0.53403 (17)	0.0868 (13)	
H18A	0.1240	0.4494	0.4869	0.130*	
H18B	0.2218	0.4112	0.5441	0.130*	
H18C	0.1566	0.4811	0.5606	0.130*	
C19	0.6290 (2)	0.30649 (14)	0.80685 (12)	0.0351 (6)	
C20	0.6729 (2)	0.29049 (15)	0.87667 (13)	0.0412 (6)	
C21	0.7816 (3)	0.31710 (18)	0.91231 (15)	0.0554 (8)	
H21	0.8107	0.3059	0.9580	0.066*	
C22	0.8483 (3)	0.36059 (18)	0.88073 (16)	0.0567 (8)	
H22	0.9203	0.3790	0.9058	0.068*	
C23	0.8093 (2)	0.37603 (16)	0.81448 (16)	0.0495 (7)	
H23	0.8551	0.4045	0.7939	0.059*	
C24	0.6985 (2)	0.34914 (15)	0.77547 (13)	0.0399 (6)	
C25	0.6616 (2)	0.36812 (15)	0.70571 (14)	0.0436 (7)	
H25	0.7135	0.3969	0.6899	0.052*	
C26	0.6419 (3)	0.2228 (2)	0.97025 (15)	0.0687 (10)	
H26A	0.6566	0.2642	1.0014	0.082*	
H26B	0.7149	0.1960	0.9758	0.082*	
C27	0.5526 (4)	0.1736 (2)	0.98454 (17)	0.0903 (13)	
H27A	0.5799	0.1554	1.0300	0.135*	
H27B	0.5386	0.1328	0.9535	0.135*	
H27C	0.4810	0.2007	0.9794	0.135*	
C28	0.2287 (2)	0.18807 (13)	0.65121 (12)	0.0308 (5)	
C29	0.1559 (2)	0.14007 (14)	0.67567 (13)	0.0363 (6)	
C30	0.0640 (3)	0.10353 (16)	0.63112 (16)	0.0535 (8)	
H30	0.0163	0.0720	0.6476	0.064*	
C31	0.0426 (3)	0.11373 (19)	0.56139 (16)	0.0644 (9)	
H31	−0.0184	0.0881	0.5319	0.077*	
C32	0.1090 (3)	0.16011 (18)	0.53657 (15)	0.0540 (8)	
H32	0.0930	0.1669	0.4901	0.065*	
C33	0.2032 (2)	0.19863 (14)	0.58073 (12)	0.0352 (6)	
C34	0.2671 (2)	0.24922 (14)	0.55117 (12)	0.0365 (6)	
H34	0.2399	0.2553	0.5046	0.044*	
C35	0.1192 (3)	0.08154 (17)	0.77209 (17)	0.0576 (8)	
H35A	0.1187	0.0336	0.7509	0.069*	
H35B	0.0388	0.0983	0.7637	0.069*	
C36	0.1749 (3)	0.0756 (2)	0.84620 (18)	0.0783 (11)	
H36A	0.1315	0.0412	0.8655	0.117*	
H36B	0.1747	0.1232	0.8668	0.117*	
H36C	0.2542	0.0586	0.8541	0.117*	
C37	0.7841 (4)	0.4555 (2)	0.2688 (2)	0.0958 (14)	
H37A	0.7900	0.4268	0.2306	0.144*	
H37B	0.8267	0.5009	0.2703	0.144*	
H37C	0.7032	0.4663	0.2647	0.144*	
C38	0.8329 (4)	0.4144 (2)	0.3298 (2)	0.0813 (12)	
Cl1	0.13472 (7)	0.44690 (4)	0.86746 (4)	0.0548 (2)	
Cl2	0.55591 (7)	0.18069 (4)	0.65509 (4)	0.05256 (19)	
Fe1	0.24653 (3)	0.35131 (2)	0.845034 (16)	0.03200 (11)	
Fe2	0.44691 (3)	0.27615 (2)	0.680267 (17)	0.03308 (11)	
H7	0.4102	0.2531	0.8409	0.050*	
H14A	0.2855	0.3687	0.6893	0.050*	
H7B	0.3042	0.2300	0.7991	0.050*	
H14B	0.3926	0.3959	0.7267	0.050*	
N1	0.8708 (4)	0.3813 (2)	0.3776 (2)	0.1248 (16)	
O1	0.37557 (14)	0.40635 (9)	0.83148 (8)	0.0357 (4)	
O2	0.33580 (16)	0.34094 (10)	0.94490 (8)	0.0422 (4)	
O3	0.17033 (14)	0.34421 (10)	0.74945 (8)	0.0368 (4)	
O4	0.12491 (16)	0.27731 (10)	0.86278 (9)	0.0421 (4)	
O5	0.09822 (18)	0.36832 (12)	0.62027 (9)	0.0562 (6)	
O6	0.51042 (16)	0.48883 (10)	0.78201 (9)	0.0417 (4)	
O7	0.33730 (15)	0.24998 (10)	0.83627 (8)	0.0392 (4)	
O8	0.31635 (14)	0.22101 (9)	0.69505 (8)	0.0335 (4)	
O9	0.35436 (16)	0.28597 (10)	0.58031 (8)	0.0403 (4)	
O10	0.52406 (15)	0.28135 (10)	0.77521 (8)	0.0376 (4)	
O11	0.56879 (16)	0.35092 (11)	0.66343 (9)	0.0446 (5)	
O12	0.59928 (17)	0.24897 (12)	0.90188 (9)	0.0515 (5)	
O13	0.18531 (16)	0.13332 (10)	0.74457 (9)	0.0420 (4)	
O14	0.35666 (15)	0.37611 (10)	0.69009 (8)	0.0391 (4)	

### Atomic displacement parameters (Å^2^)

**Table:**

	*U*^11^	*U*^22^	*U*^33^	*U*^12^	*U*^13^	*U*^23^
C1	0.0282 (13)	0.0326 (13)	0.0322 (13)	−0.0006 (10)	0.0065 (10)	−0.0087 (11)
C2	0.0313 (14)	0.0454 (15)	0.0306 (13)	0.0007 (11)	0.0070 (11)	−0.0066 (11)
C3	0.0394 (16)	0.0601 (18)	0.0342 (14)	0.0006 (14)	−0.0004 (12)	−0.0133 (13)
C4	0.0341 (15)	0.0617 (19)	0.0515 (17)	−0.0091 (14)	0.0017 (13)	−0.0206 (15)
C5	0.0368 (15)	0.0457 (16)	0.0538 (17)	−0.0092 (12)	0.0113 (13)	−0.0114 (14)
C6	0.0327 (14)	0.0365 (14)	0.0381 (14)	−0.0027 (11)	0.0100 (11)	−0.0065 (11)
C7	0.0434 (16)	0.0526 (16)	0.0235 (12)	0.0017 (13)	0.0029 (11)	−0.0024 (12)
C8	0.0502 (18)	0.0429 (16)	0.0629 (19)	−0.0088 (13)	0.0258 (15)	0.0053 (14)
C9	0.076 (3)	0.089 (3)	0.069 (2)	−0.013 (2)	0.025 (2)	0.031 (2)
C10	0.0267 (13)	0.0404 (14)	0.0352 (13)	0.0073 (11)	0.0011 (11)	−0.0065 (12)
C11	0.0287 (14)	0.0440 (15)	0.0447 (15)	−0.0002 (11)	0.0099 (12)	−0.0111 (12)
C12	0.0345 (16)	0.065 (2)	0.065 (2)	−0.0077 (14)	0.0099 (15)	−0.0148 (17)
C13	0.0355 (18)	0.108 (3)	0.067 (2)	−0.0123 (19)	−0.0041 (17)	−0.022 (2)
C14	0.0471 (19)	0.102 (3)	0.0422 (17)	0.0010 (19)	−0.0112 (15)	−0.0115 (18)
C15	0.0385 (16)	0.0628 (19)	0.0356 (14)	0.0097 (14)	0.0018 (12)	−0.0037 (14)
C16	0.0349 (15)	0.0388 (14)	0.0536 (17)	−0.0069 (12)	0.0179 (13)	−0.0040 (13)
C17	0.084 (3)	0.093 (3)	0.0333 (16)	0.032 (2)	0.0016 (16)	0.0142 (17)
C18	0.131 (4)	0.084 (3)	0.049 (2)	0.027 (3)	0.033 (2)	0.0260 (19)
C19	0.0261 (13)	0.0431 (14)	0.0335 (13)	0.0029 (11)	0.0040 (11)	−0.0105 (11)
C20	0.0327 (14)	0.0521 (16)	0.0354 (14)	0.0063 (12)	0.0038 (12)	−0.0033 (13)
C21	0.0413 (17)	0.070 (2)	0.0426 (16)	0.0059 (15)	−0.0086 (14)	−0.0056 (15)
C22	0.0320 (16)	0.069 (2)	0.060 (2)	−0.0048 (14)	−0.0034 (14)	−0.0154 (17)
C23	0.0312 (15)	0.0517 (17)	0.065 (2)	−0.0040 (13)	0.0128 (14)	−0.0137 (15)
C24	0.0312 (14)	0.0463 (15)	0.0420 (15)	−0.0013 (12)	0.0100 (12)	−0.0116 (12)
C25	0.0366 (15)	0.0462 (16)	0.0518 (16)	−0.0091 (12)	0.0187 (13)	−0.0066 (13)
C26	0.078 (3)	0.088 (3)	0.0302 (15)	0.004 (2)	−0.0023 (16)	0.0079 (16)
C27	0.109 (3)	0.119 (3)	0.0420 (19)	−0.007 (3)	0.019 (2)	0.016 (2)
C28	0.0261 (12)	0.0312 (12)	0.0340 (13)	0.0022 (10)	0.0065 (10)	−0.0069 (11)
C29	0.0313 (14)	0.0384 (14)	0.0386 (14)	−0.0006 (11)	0.0089 (11)	−0.0049 (12)
C30	0.0401 (16)	0.0514 (17)	0.067 (2)	−0.0149 (14)	0.0118 (15)	−0.0030 (16)
C31	0.0495 (19)	0.081 (2)	0.0526 (19)	−0.0257 (17)	−0.0029 (15)	−0.0149 (18)
C32	0.0459 (18)	0.070 (2)	0.0384 (15)	−0.0093 (15)	−0.0018 (13)	−0.0072 (15)
C33	0.0319 (14)	0.0409 (14)	0.0307 (13)	0.0018 (11)	0.0049 (11)	−0.0066 (11)
C34	0.0373 (15)	0.0473 (15)	0.0238 (12)	0.0091 (12)	0.0065 (11)	−0.0036 (11)
C35	0.060 (2)	0.0526 (18)	0.071 (2)	−0.0060 (15)	0.0350 (17)	0.0076 (16)
C36	0.092 (3)	0.083 (3)	0.072 (2)	0.001 (2)	0.043 (2)	0.026 (2)
C37	0.081 (3)	0.089 (3)	0.121 (4)	0.028 (2)	0.034 (3)	0.025 (3)
C38	0.080 (3)	0.071 (3)	0.097 (3)	0.023 (2)	0.031 (2)	−0.001 (2)
Cl1	0.0538 (4)	0.0557 (4)	0.0578 (4)	0.0102 (4)	0.0200 (4)	−0.0059 (4)
Cl2	0.0491 (4)	0.0603 (5)	0.0508 (4)	0.0039 (3)	0.0180 (3)	−0.0090 (4)
Fe1	0.0288 (2)	0.0411 (2)	0.02566 (18)	−0.00498 (15)	0.00670 (14)	−0.00089 (15)
Fe2	0.0304 (2)	0.0436 (2)	0.02459 (18)	−0.00648 (15)	0.00658 (15)	−0.00295 (15)
N1	0.163 (4)	0.103 (3)	0.103 (3)	0.048 (3)	0.028 (3)	0.008 (3)
O1	0.0312 (9)	0.0468 (10)	0.0276 (8)	−0.0103 (8)	0.0053 (7)	−0.0003 (8)
O2	0.0426 (11)	0.0564 (12)	0.0271 (9)	−0.0081 (9)	0.0088 (8)	0.0021 (8)
O3	0.0302 (9)	0.0504 (10)	0.0275 (8)	−0.0057 (8)	0.0042 (7)	−0.0011 (8)
O4	0.0372 (10)	0.0507 (11)	0.0387 (10)	−0.0093 (8)	0.0110 (8)	0.0018 (9)
O5	0.0547 (13)	0.0775 (15)	0.0301 (10)	0.0058 (11)	0.0012 (9)	0.0105 (10)
O6	0.0398 (10)	0.0444 (10)	0.0408 (10)	−0.0137 (8)	0.0111 (8)	0.0023 (8)
O7	0.0349 (10)	0.0467 (10)	0.0344 (9)	0.0000 (8)	0.0072 (8)	−0.0038 (8)
O8	0.0305 (9)	0.0430 (10)	0.0258 (8)	−0.0068 (8)	0.0058 (7)	−0.0036 (7)
O9	0.0410 (11)	0.0526 (11)	0.0267 (9)	−0.0066 (9)	0.0081 (8)	−0.0014 (8)
O10	0.0298 (9)	0.0538 (11)	0.0278 (9)	−0.0070 (8)	0.0054 (7)	−0.0017 (8)
O11	0.0400 (11)	0.0584 (12)	0.0362 (10)	−0.0130 (9)	0.0117 (9)	−0.0013 (9)
O12	0.0478 (12)	0.0713 (13)	0.0283 (9)	0.0010 (10)	−0.0012 (9)	0.0063 (9)
O13	0.0403 (11)	0.0453 (11)	0.0430 (10)	−0.0052 (8)	0.0159 (9)	0.0021 (9)
O14	0.0377 (10)	0.0456 (10)	0.0324 (9)	−0.0036 (8)	0.0072 (8)	−0.0051 (8)

### Geometric parameters (Å, º)

**Table:**

C1—O1	1.319 (3)	C24—C25	1.423 (4)
C1—C6	1.414 (3)	C25—O11	1.240 (3)
C1—C2	1.414 (3)	C25—H25	0.9300
C2—C7	1.416 (4)	C26—O12	1.438 (3)
C2—C3	1.420 (3)	C26—C27	1.473 (5)
C3—C4	1.353 (4)	C26—H26A	0.9700
C3—H3	0.9300	C26—H26B	0.9700
C4—C5	1.395 (4)	C27—H27A	0.9600
C4—H4	0.9300	C27—H27B	0.9600
C5—C6	1.377 (3)	C27—H27C	0.9600
C5—H5	0.9300	C28—O8	1.316 (3)
C6—O6	1.364 (3)	C28—C33	1.411 (3)
C7—O2	1.248 (3)	C28—C29	1.411 (3)
C7—H7A	0.9300	C29—O13	1.369 (3)
C8—O6	1.440 (3)	C29—C30	1.383 (4)
C8—C9	1.494 (4)	C30—C31	1.399 (4)
C8—H8A	0.9700	C30—H30	0.9300
C8—H8B	0.9700	C31—C32	1.344 (4)
C9—H9A	0.9600	C31—H31	0.9300
C9—H9B	0.9600	C32—C33	1.414 (4)
C9—H9C	0.9600	C32—H32	0.9300
C10—O3	1.315 (3)	C33—C34	1.426 (4)
C10—C11	1.401 (4)	C34—O9	1.237 (3)
C10—C15	1.422 (4)	C34—H34	0.9300
C11—C12	1.418 (4)	C35—O13	1.433 (3)
C11—C16	1.420 (4)	C35—C36	1.489 (4)
C12—C13	1.345 (5)	C35—H35A	0.9700
C12—H12	0.9300	C35—H35B	0.9700
C13—C14	1.387 (5)	C36—H36A	0.9600
C13—H13	0.9300	C36—H36B	0.9600
C14—C15	1.383 (4)	C36—H36C	0.9600
C14—H14	0.9300	C37—C38	1.436 (6)
C15—O5	1.368 (4)	C37—H37A	0.9600
C16—O4	1.241 (3)	C37—H37B	0.9600
C16—H16	0.9300	C37—H37C	0.9600
C17—O5	1.433 (3)	C38—N1	1.133 (5)
C17—C18	1.491 (5)	Cl1—Fe1	2.3007 (8)
C17—H17A	0.9700	Cl2—Fe2	2.2990 (8)
C17—H17B	0.9700	Fe1—O1	1.9088 (17)
C18—H18A	0.9600	Fe1—O3	1.9296 (16)
C18—H18B	0.9600	Fe1—O2	2.0447 (17)
C18—H18C	0.9600	Fe1—O4	2.0719 (18)
C19—O10	1.317 (3)	Fe1—O7	2.1573 (18)
C19—C24	1.408 (4)	Fe2—O10	1.9181 (16)
C19—C20	1.417 (3)	Fe2—O8	1.9343 (17)
C20—O12	1.358 (3)	Fe2—O9	2.0551 (17)
C20—C21	1.382 (4)	Fe2—O11	2.0763 (18)
C21—C22	1.396 (5)	Fe2—O14	2.1379 (18)
C21—H21	0.9300	O7—H7	0.8453
C22—C23	1.343 (4)	O7—H7B	0.8394
C22—H22	0.9300	O14—H14A	0.8504
C23—C24	1.423 (4)	O14—H14B	0.8371
C23—H23	0.9300		
			
O1—C1—C6	118.1 (2)	C26—C27—H27B	109.5
O1—C1—C2	123.4 (2)	H27A—C27—H27B	109.5
C6—C1—C2	118.5 (2)	C26—C27—H27C	109.5
C1—C2—C7	122.3 (2)	H27A—C27—H27C	109.5
C1—C2—C3	119.6 (2)	H27B—C27—H27C	109.5
C7—C2—C3	118.1 (2)	O8—C28—C33	123.3 (2)
C4—C3—C2	120.4 (3)	O8—C28—C29	118.6 (2)
C4—C3—H3	119.8	C33—C28—C29	118.1 (2)
C2—C3—H3	119.8	O13—C29—C30	125.0 (2)
C3—C4—C5	120.5 (3)	O13—C29—C28	114.7 (2)
C3—C4—H4	119.8	C30—C29—C28	120.3 (2)
C5—C4—H4	119.8	C29—C30—C31	120.3 (3)
C6—C5—C4	120.9 (3)	C29—C30—H30	119.8
C6—C5—H5	119.5	C31—C30—H30	119.8
C4—C5—H5	119.5	C32—C31—C30	120.8 (3)
O6—C6—C5	125.9 (2)	C32—C31—H31	119.6
O6—C6—C1	114.0 (2)	C30—C31—H31	119.6
C5—C6—C1	120.1 (2)	C31—C32—C33	120.3 (3)
O2—C7—C2	127.3 (2)	C31—C32—H32	119.8
O2—C7—H7A	116.4	C33—C32—H32	119.8
C2—C7—H7A	116.4	C28—C33—C32	120.1 (3)
O6—C8—C9	108.1 (2)	C28—C33—C34	122.5 (2)
O6—C8—H8A	110.1	C32—C33—C34	117.4 (2)
C9—C8—H8A	110.1	O9—C34—C33	127.5 (2)
O6—C8—H8B	110.1	O9—C34—H34	116.2
C9—C8—H8B	110.1	C33—C34—H34	116.2
H8A—C8—H8B	108.4	O13—C35—C36	108.4 (3)
C8—C9—H9A	109.5	O13—C35—H35A	110.0
C8—C9—H9B	109.5	C36—C35—H35A	110.0
H9A—C9—H9B	109.5	O13—C35—H35B	110.0
C8—C9—H9C	109.5	C36—C35—H35B	110.0
H9A—C9—H9C	109.5	H35A—C35—H35B	108.4
H9B—C9—H9C	109.5	C35—C36—H36A	109.5
O3—C10—C11	124.4 (2)	C35—C36—H36B	109.5
O3—C10—C15	117.5 (2)	H36A—C36—H36B	109.5
C11—C10—C15	118.1 (2)	C35—C36—H36C	109.5
C10—C11—C12	120.1 (3)	H36A—C36—H36C	109.5
C10—C11—C16	122.7 (2)	H36B—C36—H36C	109.5
C12—C11—C16	117.1 (3)	C38—C37—H37A	109.5
C13—C12—C11	120.5 (3)	C38—C37—H37B	109.5
C13—C12—H12	119.8	H37A—C37—H37B	109.5
C11—C12—H12	119.8	C38—C37—H37C	109.5
C12—C13—C14	120.6 (3)	H37A—C37—H37C	109.5
C12—C13—H13	119.7	H37B—C37—H37C	109.5
C14—C13—H13	119.7	N1—C38—C37	179.3 (6)
C15—C14—C13	120.9 (3)	O1—Fe1—O3	93.15 (7)
C15—C14—H14	119.6	O1—Fe1—O2	88.89 (7)
C13—C14—H14	119.6	O3—Fe1—O2	170.39 (8)
O5—C15—C14	125.8 (3)	O1—Fe1—O4	170.79 (8)
O5—C15—C10	114.4 (2)	O3—Fe1—O4	89.05 (7)
C14—C15—C10	119.8 (3)	O2—Fe1—O4	87.50 (7)
O4—C16—C11	127.9 (3)	O1—Fe1—O7	89.54 (7)
O4—C16—H16	116.1	O3—Fe1—O7	87.86 (7)
C11—C16—H16	116.1	O2—Fe1—O7	82.76 (7)
O5—C17—C18	108.2 (3)	O4—Fe1—O7	81.59 (7)
O5—C17—H17A	110.1	O1—Fe1—Cl1	99.62 (6)
C18—C17—H17A	110.1	O3—Fe1—Cl1	96.92 (6)
O5—C17—H17B	110.1	O2—Fe1—Cl1	91.99 (6)
C18—C17—H17B	110.1	O4—Fe1—Cl1	88.98 (6)
H17A—C17—H17B	108.4	O7—Fe1—Cl1	169.38 (5)
C17—C18—H18A	109.5	O10—Fe2—O8	92.46 (7)
C17—C18—H18B	109.5	O10—Fe2—O9	171.40 (8)
H18A—C18—H18B	109.5	O8—Fe2—O9	88.40 (7)
C17—C18—H18C	109.5	O10—Fe2—O11	88.82 (7)
H18A—C18—H18C	109.5	O8—Fe2—O11	170.26 (8)
H18B—C18—H18C	109.5	O9—Fe2—O11	88.92 (7)
O10—C19—C24	123.7 (2)	O10—Fe2—O14	88.45 (7)
O10—C19—C20	117.6 (2)	O8—Fe2—O14	88.73 (7)
C24—C19—C20	118.6 (2)	O9—Fe2—O14	83.01 (7)
O12—C20—C21	126.4 (3)	O11—Fe2—O14	81.66 (7)
O12—C20—C19	113.9 (2)	O10—Fe2—Cl2	97.26 (6)
C21—C20—C19	119.7 (3)	O8—Fe2—Cl2	99.93 (6)
C20—C21—C22	120.9 (3)	O9—Fe2—Cl2	91.01 (6)
C20—C21—H21	119.5	O11—Fe2—Cl2	89.47 (6)
C22—C21—H21	119.5	O14—Fe2—Cl2	169.37 (5)
C23—C22—C21	120.6 (3)	C1—O1—Fe1	131.13 (15)
C23—C22—H22	119.7	C7—O2—Fe1	126.59 (17)
C21—C22—H22	119.7	C10—O3—Fe1	129.49 (17)
C22—C23—C24	120.5 (3)	C16—O4—Fe1	124.91 (18)
C22—C23—H23	119.7	C15—O5—C17	118.1 (2)
C24—C23—H23	119.7	C6—O6—C8	117.2 (2)
C19—C24—C25	122.6 (2)	Fe1—O7—H7	116.9
C19—C24—C23	119.6 (3)	Fe1—O7—H7B	108.8
C25—C24—C23	117.8 (3)	H7—O7—H7B	109.8
O11—C25—C24	127.9 (3)	C28—O8—Fe2	129.83 (15)
O11—C25—H25	116.1	C34—O9—Fe2	125.71 (17)
C24—C25—H25	116.1	C19—O10—Fe2	129.90 (16)
O12—C26—C27	108.4 (3)	C25—O11—Fe2	124.78 (18)
O12—C26—H26A	110.0	C20—O12—C26	117.8 (2)
C27—C26—H26A	110.0	C29—O13—C35	117.1 (2)
O12—C26—H26B	110.0	Fe2—O14—H14A	112.6
C27—C26—H26B	110.0	Fe2—O14—H14B	107.6
H26A—C26—H26B	108.4	H14A—O14—H14B	109.9
C26—C27—H27A	109.5		

### Hydrogen-bond geometry (Å, º)

**Table:**

*D*—H···*A*	*D*—H	H···*A*	*D*···*A*	*D*—H···*A*
O7—H7···O10	0.85	2.22	2.887 (2)	136
O7—H7···O12	0.85	2.25	3.027 (3)	153
O14—H14*A*···O3	0.85	2.13	2.862 (2)	145
O14—H14*A*···O5	0.85	2.28	3.008 (3)	143
O7—H7*B*···O8	0.84	2.19	2.896 (2)	142
O7—H7*B*···O13	0.84	2.33	3.063 (2)	146
O14—H14*B*···O1	0.84	2.23	2.908 (2)	139
O14—H14*B*···O6	0.84	2.28	3.026 (2)	149
C7—H7*A*···Cl2^i^	0.93	2.80	3.724 (3)	171
C34—H34···O2^ii^	0.93	2.57	3.014 (3)	110
C37—H37*C*···O6^iii^	0.96	2.58	3.506 (5)	162

Symmetry codes: (i) *x*, −*y*+1/2, *z*+1/2; (ii) *x*, −*y*+1/2, *z*−1/2; (iii) −*x*+1, −*y*+1, −*z*+1.
